# Executive Functions in Decision Making under Ambiguity and Risk in Healthy Adults: A Scoping Review Adopting the Hot and Cold Executive Functions Perspective

**DOI:** 10.3390/brainsci12101335

**Published:** 2022-10-02

**Authors:** Laura Colautti, Alessandro Antonietti, Paola Iannello

**Affiliations:** Department of Psychology, Catholic University of the Sacred Heart, 20123 Milan, Italy

**Keywords:** decision making, Iowa Gambling Task, Game of Dice Task, executive functions, cognition, healthy adults

## Abstract

Decision making (DM) has a pivotal role in supporting individual autonomy and well-being. It is considered a complex ability exploiting many cognitive functions, among which executive functions (EFs) are crucial. Few studies analyzed the role played by EFs in DM in healthy adults under ambiguity and risk, which are common conditions for most decisions in daily life. This scoping review aims to analyze the relationships between two individual tasks widely used to assess DM under these conditions (Iowa Gambling Task and Game of Dice Task) and EFs. According to the organizing principle that conceptualizes hot and cold EFs, DM under such conditions mainly implies hot EFs, but the relationship with cold EFs is still unclear. Using such an approach, a comprehensive framework is provided, highlighting main findings and identifying possible gaps in the literature. The results suggest different roles played by cold EFs in DM under ambiguity and risk, according to the characteristics of the tasks. The findings can offer guidance to further studies and to design interventions to support DM in healthy adults.

## 1. Introduction

Decision making (DM) is pervasive in everyday life, covering several areas of people’s activities (i.e., medical, economic, affective, and working ones). Hence, it is considered a crucial ability to support individual well-being and autonomy [1,2,3].

DM can be conceptualized as a goal-directed multistep process (e.g., see the multistep decision model [4,5]) that involves both cognitive and affective components. First, an evaluation process usually occurs, in which a key role is played by the individual’s motivation to establish whether personal needs are satisfied. This evaluation can be driven by a modification of the external environment (e.g., something of interest is noted by the subject) and/or of the internal states (such as feeling a particular emotion). If the individual judges that he/she needs to modify the current state, the next step involves the selection of the most opportune goal among several alternatives that differ in one or more characteristics. The selection occurs by setting values to each alternative and by adjusting them according to the contingent context. Then, the evaluation of a strategic plan for achieving the selected goals with higher probabilities occurs and the best way to achieve the goal is planned. A final evaluation needs to be conducted to establish whether something in the environment and/or in the internal status has changed in the meantime. Therefore, if something has impacted previous evaluations and a large mismatch between the predicted result and the goal occurs, the individual inhibits the planned actions. Conversely, if the planned actions are appropriate, the actions are executed by the individual, who stores the results of the made choices. In this process a crucial role is played by computing predictions concerning future rewards and by estimating the discrepancy between actual and expected outcomes. Feedback is used to update decisional representations that can be useful to optimize future choices [4,5,6]. Thus, making a “good” decision involves the selection of the option that minimizes possible costs and maximizes advantageous consequences or, from a broader point of view, leads to the best result in terms of “biological fitness” [4,5,7,8].

In most cases making a decision means reasoning under conditions of uncertainty, as it is not possible to predict with certainty the final outcome or the consequences of the alternatives. A distinction based on the probability of the outcomes can be made between DM under ambiguity and under risk. The former includes situations of uncertainty in which the probability of positive or negative outcomes, associated with at least one of the possible choice options, is unknown. Whilst the latter includes those situations in which the probabilities of the occurrence of possible outcomes are known and more data can be considered in the decisional process [9,10,11].

Focusing on a cognitive point of view, to make a decision several steps are required, which may be biased by emotions elicited by the contingent situation: recognizing the current situation and identifying crucial information, integrating and updating available information, evaluating and comparing possible outcomes of the choice options according to the personal goals and motivation, inhibiting impulsive responses, anticipating the positive or negative consequences of possible choices (according to the data available and feedback derived from previous similar situations), making a decision according to the contingent personal goals, and re-evaluating the decision according to the outcome (e.g., [12]).

Several studies—most of which investigated behavioral outcomes in neurological patients rather than in healthy samples—pointed out that DM under ambiguous and risky conditions may be related to executive functions (EFs), although so far it is not entirely clear how and which EF abilities are involved in DM under such conditions (e.g., [13,14,15,16,17]. Considering the possible detrimental consequences in everyday life derived from poor decisions, it appears crucial to gain a deeper understanding of the role played by EFs in DM under ambiguity and risk in healthy adults. This can lead to the identification of possible knowledge gaps for further research, with the aim to design both effective ways to support individuals’ decisional process and more sensitive tools in the early detection of decisional impairments.

### 1.1. A Conceptualization of Executive Functions

EFs are often defined as an “umbrella term” that includes a wide set of high-level cognitive abilities that are goal-directed and future-oriented (such as sequencing, planning, using feedback, cognitive flexibility, and resistance to interferences) [18,19]. Many authors over time have tried to create models that could describe EFs to offer indications on how to investigate and assess them. In fact, the existence of a single underlying ability or dimension that can encompass all the EF components was first assumed (such as [20,21,22]), although to date there is a fair agreement to consider EFs in adulthood as organized in separable cognitive components [23,24]. In this way, one of the most considered frameworks was proposed by Miyake and colleagues [18], in which three distinguishable but moderately correlated core abilities are identified, representing fundamental and specific processes: *inhibition* of prepotent responses, information *updating* and monitoring, and mental set *shifting*. Additionally, Diamond [25] highlighted three core EFs: inhibition (behavioral inhibition and interference control), working memory, and cognitive flexibility. From such core abilities, higher-order EFs are set up, such as reasoning, problem solving, and planning. Diamond’s conceptualization differs from Miyake and colleagues’ one for mainly two characteristics. First, Diamond assumed that the core EFs work synergically to set up the other higher-order EFs (i.e., reasoning, problem solving, and planning). Second, there is no underlying or common unitary mechanism, contrary to what Miyake and colleagues argued [24].

Recently, it has been claimed that emotions and motivation can interact with EFs at both a cognitive and a neural level, affecting (positively or negatively) the behavioral response. The effects of cognition and emotions can be interpreted as an integration between these two aspects [26,27]. This assumption highlights the importance of considering the affective aspects involved in a task. It is worth mentioning an organizing principle that accounts also such aspects, namely, conceptualizing EFs as along a continuum from *cold* EFs to *hot* EFs, based on the extent they imply emotions [28,29,30]. In this way, *cold* EFs involve those processes which are (relatively) “purely” cognitive, as they are assessed in emotionally neutral contexts. Therefore, they are behaviorally investigated through tasks that generally do not underly an affective component and involve skills such as planning, set-shifting, working memory (or updating), and inhibition. In contrast, *hot* EFs are involved in situations characterized by the presence of a motivational component and imply the processing of the incentive value and the reversal of approach-and-avoidance tendencies [30,31,32,33]. In recent times, the usefulness of adopting this approach has been highlighted because (i) both the affective and cognitive dimensions are considered, (ii) their implication depends on contextual information, and (iii) more inclusive brain areas are considered for EFs [33]. Neuroimaging studies pointed out several brain areas that underlie cold and hot EFs, in which a crucial role is covered by the prefrontal cortex (PFC). In fact, cold EFs are supported by a cognitive control network including the lateral portion of PFC (lPFC), dorsal anterior cingulate cortex (dACC), and parietal cortex, in which the dorsolateral prefrontal cortex (dlPFC) is pivotal [33,34,35]. In hot EFs cognitive operations which are emotionally charged are associated with medial, ventromedial, and orbital portions of the PFC (respectively, mPFC, vmPFC, and OFC), which relate to subcortical structures involved in motivation and emotions, such as insula, amygdala, and the limbic system [36,37]. Furthermore, in emotionally connoted situations that require control processing, not only mPFC and OFC but also lPFC and anterior cingulate cortex (ACC) are involved [26,33,38].

According to the conceptualization of hot and cold EFs, both DM under risk and ambiguity can be considered as mainly belonging to hot EF components, as DM in such conditions is rarely characterized by the absence of emotional or motivational aspects [33,39]. Nevertheless, the relationship with cold components of EFs—and, specifically, when and how they intervene in the DM process—is still unclear.

### 1.2. Cold and Hot Executive Functions: What They Tell Us about Decision-Making Abilities

It is assumed that the involvement of hot and cold EFs is based on the nature of the task, namely, on the degree of the involvement of cognitive control and emotional aspects [40]. This is in line with neuroimaging studies that showed overlaps between neural areas involved during tasks that mainly imply cold EF components and those that require making a choice under ambiguous and risky conditions (which encompasses hot EFs). Specifically, DM processes under these conditions are supported by cortical and subcortical neural networks in which an important role is played by the PFC (and the so-called frontostriatal loops) and by dopamine, a neurotransmitter fundamental for reward processing, motivation, and learning [14,41,42]. In particular, dlPFC, mPFC, and ACC are involved in error-detection ability and in reward/risk processing linked to the midbrain dopamine [43,44]. Moreover, a crucial role in DM is played by basal ganglia (BG), vmPFC, ventrolateral PFC (vlPFC), and OFC, which are involved in the processing of feedback, positive outcomes (or rewards), and emotionally charged events, and in the ability to anticipate consequences [45,46,47].

Consistently with the possible interplay of cold and hot EF components in DM, it is worth mentioning the model proposed by Brand and colleagues [14]. It is assumed that there are two ways (or routes) for making decisions. One involves non-declarative knowledge that underlies emotions experienced as a consequence of previous positive vs. negative feedback. Emotions may affect the decisional process, relying on a sort of “gut feeling”. On the other hand, the other route mostly implies cognitive components. It is assumed that working memory is essential, as it allows the decision-maker to elaborate available information, to compare the contingent situation with previous similar ones, and to form, monitor, and—if optimal—maintain a decisional strategy. Furthermore, other EF components, such as categorization and flexibility (or shifting), allow the recall of previously experienced situations from long-term memory. According to this model, while decisions under ambiguity mainly involve the first route, as assumed by Bechara and colleagues [48] and, according to the hot and cold EF conceptualization, mostly rely on hot EFs (and in a lower degree on cold EFs), in risky conditions the decision-maker rely (also) on the other route or integrate both, involving cold EFs in a higher degree.

### 1.3. Decision Making in Healthy Adulthood

Several studies showed that, compared to younger adults (YAs), older adults (OAs) exhibit more difficulties in performing tasks requiring numerical operations, applying high-cognitive load reasoning, and choosing an adequate decisional strategy relying more on emotional aspects and being more affected by DM biases. Such differences are more marked in unusual situations (where previous experiences or crystallized intelligence have a minor role) and under time pressure (e.g., [44,49,50,51,52,53]).

Moreover, OAs usually present a decrease in anticipation of loss but not of gain in DM under ambiguous and risky conditions, if compared to YAs [54,55]. Neuroimaging studies which investigated responses in monetary tasks outlined that both OAs and YAs show an increased activity in the nucleus accumbens in anticipating possible gains, whereas only YAs present an increased activity in the anterior insula in loss anticipation [55]. Skin conductance responses (SCRs) highlighted that OAs who make more functional decisions present discriminatory anticipatory SCRs, in which positive results, rather than negative ones, support better choices. In contrast, OAs who perform worst do not discriminate between positive and negative results, presenting similar anticipatory SCRs [56]. Therefore, it can be assumed that, although YAs make better decisions depending more on the anticipation of negative outcomes, OAs based them mostly on the anticipation of positive outcomes, which may lead them to underestimate losses and make riskier (and poorer) choices than YAs. This orientation toward positive emotions is consistent with studies highlighting that OAs are more focused on positive emotions, experiencing lower levels of negative emotions but similar levels of positive ones than YAs (e.g., [57,58,59]). Thus, a decrease in the quality of decisions under ambiguity and risk may occur with physiological aging, so that OAs, compared to YAs, tend to be more risk-takers, mostly seeking positive outcomes or gains, rather than avoiding negative outcomes or losses.

Age differences in DM are probably due to cerebral changes occurring during the lifespan, as a volumetric decrease of gray and white matter, especially in superior and anterior cortical regions (mainly in the frontal, prefrontal, and temporal cortex [60]) and to a decline in dopamine modulation, involving frontostriatal network functioning [55], which is implied both in decisional processes and in cold EFs. In addition, probably due to the cerebral changes previously described, impairments in cold EF components—which generally occur in the aging process (e.g., [61])—can affect the decisional process, especially in tasks involving cold EFs to a greater extent.

It appears crucial to delve into the relationship between DM and cold EF components to understand how decisional abilities can be supported for preventing impairments or loss of autonomy along the lifespan. This appears important especially in the later stages of life when consequences derived from poor choices can have a greater and detrimental impact on well-being. The results in literature about the relationship between EFs and DM under ambiguity and risk during the lifespan are sometimes inconsistent. The controversial findings are at least in part due to the multitude of tasks used to assess DM under conditions of ambiguity and risk. Generally, these tasks require the elaboration of monetary gains and losses and the decision-maker must choose between two or more options (such as the Balloon Analogue Risk Task [62], the Columbia Card Task [63], the Iowa Gambling Task [64,65], and the Game of Dice Task [66]). However, such tasks involve cold EFs in a different way and include ambiguity-and-risk components in a different extent, making comparisons difficult [67]. Moreover, some of them are very often applied in single-case studies (e.g., [68]), so that the possibility to generalize is reduced.

### 1.4. Aims

According to the purposes of a scoping review (e.g., [69]), the present article aims to explore emergent evidence in literature to provide a comprehensive overview of the relationship between DM and cold EFs in healthy adults, considering the most frequently used tasks in literature to assess DM under ambiguity and risk (for more details, see Section 2.2). It is worth specifying that the hot and cold EF conceptualization has not commonly been adopted in the literature, but we argue that it can represent a useful approach for analyzing decisional processes as it considers both the cognitive and the affective aspects involved in a task. Therefore, the goal is to clarify the role of EFs in DM according to such an organizing principle and to investigate the correlations between cold EFs and the behavioral parameters used in the selected papers.

Specifically, we focused on the relationship between DM under ambiguity and risk and cold EF components for two main reasons: (1) While there is a consensus that DM under ambiguity and risk encompasses hot EFs (e.g., [33,39]), the relationship with cold components of EFs is still confused; (2) It appears crucial to understand how those abilities that are mainly cognitive (or cold) can affect the quality of decisions and to further understand how to support DM process and sustain the individual autonomy.

Moreover, the present scoping review aims at identifying possible knowledge gaps and further research directions to better understand decisional mechanisms.

## 2. Materials and Methods

The present scoping review was conducted according to the Preferred Reporting Items for Systematic Reviews and Meta-Analysis Extension for Scoping Reviews (PRISMAScR) [70]. Differently from systematic reviews, a registered protocol is not needed for scoping reviews [69]. The five-stage framework [71] was also considered to develop the methodology of the present scoping review, adopting a process that allows for the replicability of searching strategies, increases the reliability of the findings, and ensures transparency. In detail, the five stages are (1) identifying the research question; (2) identifying relevant studies; (3) study selection; (4) charting the data; and (5) collating, summarizing, and reporting the results.

### 2.1. Identifying the Research Question (Stage 1)

The questions which guided the present scoping review were as follows: Is there a correlation between cold EF components and DM under ambiguity in healthy adults? Is there a correlation between cold EF components and DM under risk in healthy adults? Considering possible differences in cold EFs due to aging, does age have a role in the relationships between DM and cold EFs?

### 2.2. Identifying Relevant Studies (Stage 2)

To investigate DM under ambiguity and risk, we considered the Iowa Gambling Task (IGT) [64,65] and the Game of Dice Task (GDT) [66] (for a description of the tasks, see Appendix A). This choice was made on the basis of three considerations: (1) They are the two most used tools requiring an individual administration [72]; (2) They mirror situations of ambiguity and risk, which are well recognized by literature; (3) They focus only on the relevant aspects of DM, and thus they limit, as far as possible, any confounding variability in the results.

The search was updated on 1 March 2022. It included articles published since 2000 in peer-reviewed journals indexed in PsycINFO, PubMed, and Scopus. According to the aims (for more details, see Section 1.4), the keywords entered for the search were “Iowa Gambling Task AND (executive functions) AND (adults)”, “Game of Dice Task AND (executive functions) AND (adults)”. After the study selection (Stage 3, see below), bibliographies of the selected studies were checked to include other possible eligible studies.

Then, to decide which studies to keep, the following inclusion criteria were applied: (1) Studies recruited healthy adults from 18 years old; (2) All the participants of the studies did not present any neurological, behavioral, or psychiatric disease; (3) Studies addressed the relationships between the outcomes of the decisional tasks and cold EFs, through the administration of validated instruments to assess the latter ones; (4) The standard, but not modified, versions of the tasks were employed; (5) Papers were written in English.

The exclusion criteria adopted were as follows: (1) Participants presented neurodegenerative, autoimmune, or systemic diseases (e.g., Alzheimer’s disease, Parkinson’s disease, multiple sclerosis, lupus, HIV, or thyroid disorders), cognitive impairments (e.g., dementia, severe single/multiple cognitive domain impairments, or learning disability), behavioral or psychiatric disorders (e.g., substance/alcohol abuse, obsessive compulsive behaviors, major depression, eating disorders, or schizophrenia), or altered states (e.g., sleep deprivation, intake of soft drugs before the assessment, or concomitant tasks that induce stressful conditions); (2) Participants underwent cranial surgery; (3) Participants underwent cognitive training; (4) Studies aimed to validate an instrument without analyzing the relationship between DM and cold EFs through standardized tests; (5) Book chapters.

### 2.3. Study Selection (Stage 3)

**Figure 1:**
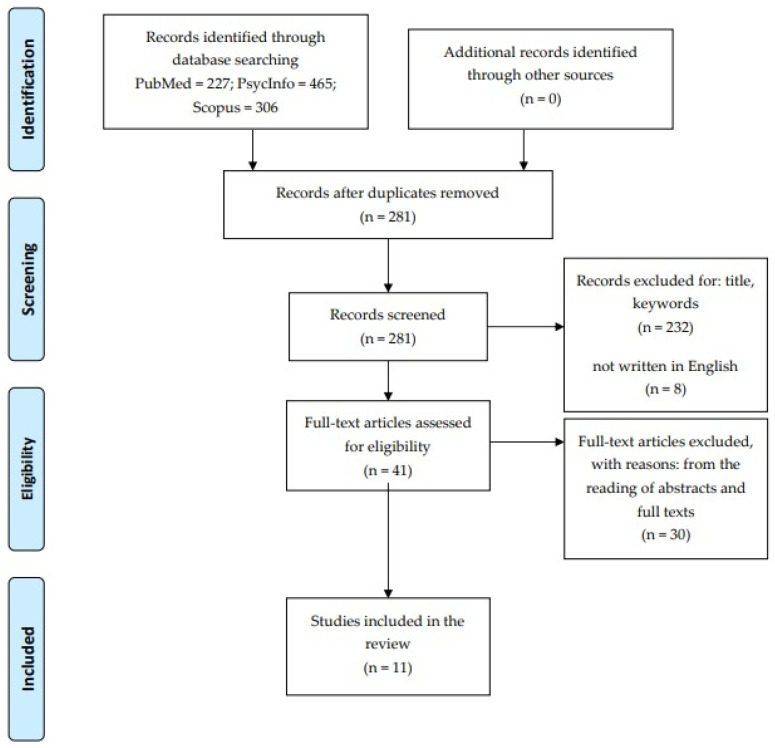
PRISMA flow diagram for studies considered in the scoping review.

L.C. screened the relevant articles, first by title, keywords, and language and then by reading the abstracts and full texts. The selection of studies followed the Preferred Reporting of Items for Systematic Reviews and Meta-Analyses (PRISMA) Statement [73] (see Figure 1 for more details). Possible doubts about the inclusion of the studies were discussed with P.I. and A.A. to reach a consensus.

### 2.4. Charting the Data (Stage 4)

An electronic form was designed to decide which variables to consider. Extracted data followed, where possible, Arksey and O’Malley’s [71] recommendations to make comparisons between studies. Reported data regarded authors and year of publication, country where the study was conducted, size of the sample, sample’s age and level of education, considered variables of the decisional tasks, assessed cold EFs and other cognitive functions, assessment tools, and main results.

### 2.5. Collating, Summarizing, and Reporting the Results (Stage 5)

An analytic framework was considered to present a narrative report of existing literature following the PRISMA guidelines—extension for scoping review [70].

## 3. Results

### 3.1. Characteristics of the Studies

A total of 11 studies had been selected (see Figure 1), among which six investigated the IGT [10,74,75,76,77,78] and five investigated the GDT [79,80,81,82,83]. Concerning those that investigated the IGT, five studies were conducted in the USA and one was conducted in China, whilst all the studies administered the GDT were conducted in Germany.

Regarding the IGT, participants’ lowest average year was 20.82 years [78] and the higher one was 54.70 years [76]. In contrast, regarding the GDT, participants’ lowest average year was 23.64 years [81] and the higher one was 44.45 [79] (for more details, see Table 1).

### 3.2. The IGT and Cold EFs

Six studies have been considered [74,75,76,77,78,79]. As reported in Table 2 and Table 3, four of them [10,75,76,78] assessed cold EFs also through the Wisconsin Card Sorting Test (WCST; [84]) or the Modified Card Sorting Test (MCST; [85]). In detail, these tests investigate the ability to categorize and shift from an old strategy to a new one when external feedback seems no longer to be valid [18,86]. In Fein and colleagues [74]’s study, cold EFs were assessed through a plethora of neuropsychological tests, divided into two cognitive domains: abstraction-flexibility and working memory. For each of them, the age-adjusted Z scores were computed. In Suhr and Hammers’ [75] and Xue and colleagues’ [77] studies cold EFs were also investigated through a working memory task, shaped according to the standard paradigm of the N-back task (see Table 2 and Table 3 for more details).

In Brand and colleagues’ [10] study, consistently with the literature, a detailed analysis of IGT performance was carried out for each block composed of 20 trials. The authors found that, in trials 1–20, the choices were riskier (and so, more disadvantageous); In trials 21–40 there was a shifting in choices, where participants started to choose more advantageous decks (which are safer); Finally, in trials 41–100 the choices were clearly advantageous. The authors found a significant relationship between IGT and WCST only in IGT trials in which the risk component gradually appears over the ambiguity component, namely, in trials 21–100. Specifically, the relationship was stronger in trials 81–100 (the final ones), in which the participant should have definitively identified the advantageous and disadvantageous decks, and so the probability of win-and-loss occurrence should be more explicit.

Similarly, also in Gansler and colleagues’ [76] study, the IGT performance was divided into blocks. A structural equation modeling was performed, from which a relationship between the decisional performance and attention in the whole task and with cold EFs (assessed through MCST) emerged in trials 41–100, supporting the possible role of EFs when the rules of the task become more explicit for the participant, and so there are more data to make the decision.

Fein and colleagues [74] compared YAs to OAs (with a breakpoint of 55 years old), showing different relationships between IGT and cold EF components. Specifically, in OAs correlations were found with abstraction and flexibility as well as with immediate memory. In YAs correlations emerged with working memory as well as with psychomotor ability. Furthermore, the authors investigated possible differences in DM through the IGT during the lifespan, showing that OAs made more disadvantageous choices than YAs.

Interesting results emerged from Suhr and Hammers’ [75] study as well. In detail, they divided participants into two groups according to their performance in the IGT: “failures”, the group obtaining low scores in the task, and “passers”, the group achieving high scores according to normative data [65]. The two groups did not differ in sociodemographic characteristics, personality traits, or WCST errors. A significant difference between the two groups was found in nonverbal reasoning and in working memory, specifically in commission errors (namely, false-positive errors, consisting in choosing an item when it is not appropriate), which imply a component of impulsivity (and so, showing possible difficulties in inhibiting an answer). For the latter test—the results of which should be considered with caution given the non-standardized nature of the assessment—a relationship between the performance in the decisional task and the updating ability emerged, more specifically that underlying inhibition, an ability which is needed by the task. Moreover, using the Near-InfraRed Spectroscopy (NIRS) technique, the authors found that the “failures” group presented less bilateral dlPFC oxygenation than the other group. Furthermore, the group who failed the IGT exhibited a significant reduction in right frontal cerebral activation. These data may sustain the claim that cold EF components—such as updating, which is usually linked to lPFC [87]—are crucial in IGT.

No relationship between the IGT performance and cold EFs emerged both in Xue and colleagues’ [77] and in Reynolds and colleagues’ [78] studies.

### 3.3. The GDT and Cold EFs

Considering DM under explicit risk, as assessed by the GDT, five studies have been analyzed [79,80,81,82,83]. All the studies reported a correlation between the GDT and cold EFs (for more details, see Table 2 and Table 3). Brand and colleagues [79] investigated also the strategy used to perform the GDT (i.e., whether the choice in each trial was made after mathematical reasoning (using a deliberative approach) or was based on intuition (intuitive approach, namely, the opposite of the deliberative approach)). The results showed that the more a deliberative approach was used, both the higher the GDT net score was and the more frequently advantageous choices were made, whilst the more an intuitive approach was adopted, the more frequently risky choices were made. In addition, the tendency to adopt a deliberative approach was associated with cold EFs and logical reasoning, while the tendency to rely on an intuitive approach did not show correlations with those measures. Hence, it appears that adopting an approach relying to a greater extent on “cognitive” abilities to make a decision is more effective in making functional and non-risky choices and it is related to good levels of cold EF components. Conversely, relying mainly on an intuitive approach leads to more disadvantageous decisions. In addition, Schiebener and colleagues [80] reported that the abilities to categorize and be flexible contributed to predicting the choices in the GDT. Specifically, those who made more advantageous and non-risky choices in the task presented a better performance in the MCST. The same was true for participants with a better performance in logical reasoning. In Schiebener and colleagues’ [81] study, the authors explored the effects of possible contextual variables, such as goal (participants had to set the winning amount to be reached by themselves) and anchor (participant was presented a so-called “Top 10 list”, in which the higher amounts ever won, characterized by the possible but improbable high values, were reported). Hence, it probably leads the decision-maker to overestimate the probability of very high wins and so the low occurrence of losses. The results highlighted that—independently from the presence of contextual variables of influence—people with higher functioning in cold EF components performed better in the GDT. On the other hand, participants with lower functioning in cold EF components performed worst when they were exposed to a goal or an anchor. It indicates that EFs may influence the elaboration of available data and the resulting reasoning, leading to advantageous choices.

Two studies investigated possible differences in aging [82,83]. Brand and Schiebener [83] reported that age was a predictor of GDT performance. The authors found that older participants performed worse in the GDT, but this relationship was moderated both by logical reasoning and cold EFs. More specifically, older participants with a better performance in cold EF components made less risky choices than peers with a lower performance in cold EF components. Furthermore, Schiebener and colleagues’ [82] results confirmed the negative relationship between age and GDT performance. In addition, they found that the abilities to inhibit, control interference, and shift mainly predicted the performance in GDT and mediated the influence of the other abilities which were assessed.

## 4. Discussion

Regarding DM under ambiguity, in four out of six studies a relationship between the IGT and cold EF components emerged. Studies that delved into the DM performance by dividing the IGT into blocks [10,76] showed stronger correlations between cold EFs (investigated through WCST or MCST) and the second part of the IGT. Such data can support that hot EFs and cold EFs work synergically, along a continuum, according to the contingent situation, consistently with findings from other studies (e.g., [27,88,89,90]). The involvement of cold EF components, also highlighted by evidence regarding the pivotal role of the dlPFC in performing the IGT [75], may depend on the nature of the cognitive demands—which dynamically involve different degrees of cognitive control on affective aspects—as the decision-maker gains more experience (and more information) about the task. Accordingly, it is worth mentioning results from the administration of the GDT, in which (in conditions of risk) all information is given from the beginning of the task. In all the studies considered the performance in the GDT was related to cold EF components, and in particular to the abilities to shift and be flexible, to categorize, to inhibit inappropriate or automatic responses, and to monitor. In this regard, cold EF components represent a crucial resource to making advantageous choices, allowing the decision-maker to elaborate all available information and make less risky choices, even if the context induces biases that can negatively affect the decisional process (e.g., negative contextual influences [81] or absence of feedback [91]). This conclusion can also be a further support in considering that cold EFs and hot EFs synergically operate to ensure more adaptive responses.

### 4.1. A Critical Analysis of the Results and Possible Further Directions

#### 4.1.1. The Possible Role of Other Variables

Good levels of cold EF components could be a resource for taking optimal and not risky choices [79]. However, other variables can sustain DM and deserve to be delved into in further studies. From the present scoping review, logical reasoning (often linked to DM performance in the analyzed studies) merits being taken into account. It can be encompassed in fluid intelligence abilities, considering both (i) a possible definition of them as skills important to reason, to solve novel problems, and to learn quickly from experience, and (ii) the possible ways of measuring them, generally using problem-solving tasks requiring the generation and verification of hypotheses [92], and so overlapping logical abilities. Accordingly, studies often showed an unclear relationship between cold EFs and reasoning because the two constructs present similarities, being both core aspects of intelligence and being both associated with frontal lobe functioning [93]. Further studies might better clarify the relationship between them, contributing to delving into the decisional process and its relations with cold EF components. Moreover, from the analyzed studies it emerged that crystallized intelligence is not associated with DM under ambiguity or risk. We can suppose that, at least to some extent, these results can be attributed to the different nature of the stimuli (mainly verbal for assessing crystallized intelligence vs. also figural for assessing decisional tasks) and, mostly, to the underlying cognitive operations (i.e., involvement of knowledge and experiences previously learned during the lifespan (crystallized intelligence) vs. analyzing new situations, identifying the underlying mechanisms through received feedback, and finding functional strategies and responses for decisional tasks).

Moreover, concerning studies investigating IGT, a question might arise: Could a different result have emerged if all the considered studies had analyzed IGT performance by dividing it into at least two parts (e.g., the prior one, where conditions of ambiguity are mainly present, and the subsequent one, where the performance is affected also by a component of risk)? In the four studies that had not explored possible differences in IGT performances across the blocks (unlike the two studies which analyzed it), we can speculate that results from the first part of the task, in which the decision is close to the random level, may have weakened or biased a possible relationship with cold EFs. Therefore, further studies investigating the relationship between cold EF components and the performance in each block of the IGT are needed to better understand cognitive processes that occur during the task and to clarify whether cold EFs contribute to modifying (or modulating) the decision strategy along the task, moving from a situation of ambiguity to one of risk.

Accordingly, the model described in the prior paragraphs about the two possible routes to make decisions seems to be relevant to analyze the decisional process in the IGT. When ambiguous conditions are predominant (first part of the IGT), cognitive abilities (e.g., cold EF components) seem to have a minor role in making decisions, suggesting that other aspects may intervene, such as individual differences or emotions triggered by feedback, leading to a more “gut-oriented” choice. In this way, a relationship emerged between the IGT and the propensity for risky behaviors, in which lower levels of cold EFs increase the probability of engaging in risky behaviors [78]. According to previous statements, these results may be seen as an ineffective “modulation” of cold EFs on hot EFs processes, which may be predominant in these circumstances. Furthermore, in Xue and colleagues’ [77] study, another personal difference seems to be negatively related to the IGT performance: the gambler’s fallacy, which represents the tendency to believe that a randomly occurring event is less probable after a series of the same events. The presence of such a fallacy is probably due to a poor balance of cognitive (cold components) and affective (hot components) decisional mechanisms. Hence, it seems that, when information is lacking, individual characteristics may compensate for this lack, playing a greater role in the decisional process. Delving into individual differences can contribute to shedding light on the inconsistency of results about the relationship between the IGT and cold (and hot) EF components.

#### 4.1.2. The Effect of Age on DM

It is worth mentioning an interesting aspect that emerged in three studies (one administering the IGT [74] and two the GDT [82,83]), namely, the effects of age. Specifically, the first-mentioned study seems to support the presence of possible impairments in decisional abilities in OAs if compared to YAs, in which cold EF components may act as a protective role for the elderly (focusing on positive correlations found by authors between the IGT net score and levels of abstraction and flexibility in OAs). In making decisions under risk, the performance is negatively related and predicted by age, but the relationship is moderated by cold EFs. This is consistent with literature that highlights differences during the lifespan in the application of functional strategies and in feedback processing about rewards and punishments (e.g., see [56,94]). This is also consistent with other studies that showed in OAs the tendency toward making risky choices (e.g., [54,55]. Accordingly, DM between YAs and OAs can underlie cerebral changes occurring in aging (as specified in the Section 1). Referring to studies that pointed out possible differences in DM between YAs and OAs and considering the demographic characteristics of the samples recruited in the studies analyzed in the present review (Table 1), we can observe that in some of them participants belonged to broad and heterogeneous age groups, whose performance was analyzed aggregately. Further studies are needed, both to replicate results derived from the three studies that investigated the effect of aging on the relationship between DM and EFs and to avoid possible biases due to the lack of distinction between clear age groups.

#### 4.1.3. The Assessment of Cold EFs

Finally, analyzing the tools used to assess cold EFs in the studies, the crucial issue of the assessment of cold EFs should be considered. To grant replicability of the results, it would be preferable that future studies will administer the tools so far mostly used—namely, WCST or MCST—to allow researchers to make comparisons between studies. Conversely, it can be reductive to assess EFs based only on “complex tests”, which underlie more simple cognitive abilities, as it happens for WCST or MCST. In fact, the risk with those tests is to underspecify the specific abilities involved [18], and so the role played by these abilities cannot be independently evaluated. Thus, it is preferable to assess cold EFs by examining in a more specific way their main and basic components. For instance, considering the work led by Miyake and colleagues [18] or by Diamond [25], three distinguishable core abilities are identified, which represent fundamental and specific cognitive abilities: inhibition, updating/working memory, and shifting/flexibility. Accordingly, referring to the studies considered in the present review which have analyzed these components in a targeted way, the results are significant and deserve to be deepened in future research.

#### 4.1.4. Possible Implications

The present scoping review would contribute to a deeper understanding of the mechanisms that underlie DM offering a theoretical framework for analyzing the findings reported in the literature and delving into cold EF components involved in the DM process. Several practical implications can follow. First, the results can be a starting point for researchers and clinicians to promote further studies on the issue by investigating the open questions still existing in the literature. Second, results may be useful for designing new instruments addressed to better assess DM under ambiguity and risk. Third, further research could verify if the presence of impairments in cold EF components related to the decisional process—which are commonly assessed in clinical practice—could be an early indicator to detect those people who may present or are developing initial difficulties in DM, preventing possible negative consequences in their daily life. Moreover, such data can contribute to developing the theoretical bases for designing cognitive programs to effectively support and rehabilitate DM through the enhancement of cold EFs. This could be possible by providing structured and adaptive cognitive training based on exercises involving cold EF components such as shifting, updating/working memory, and inhibition. So far, it was found that fostering cold EF components can improve decisional abilities [95,96], even if more studies are needed to better clarify such results. Finally, from a broader point of view, knowing the mechanisms involved in DM under ambiguity and risk can offer healthcare providers and public stakeholders useful information for communicating data involving risky choices that can sustain the decisional process, especially in the later stages of life (e.g., starting or changing therapeutic plans; investing savings in one solution rather than another).

### 4.2. Limitations and Strengths

The present scoping review presents some limitations, mainly due to the features of the existing literature. First, the examined studies (which were restricted in number) were rather heterogeneous due to different designs, methodologies used to collect and analyze data, and instruments chosen to assess cold EFs. Second, when the methodology used was similar, as happened in studies that investigated GDT, they were conducted in the same geographical zone (Germany). Therefore, it is difficult to define the replicability of the results. Third, other publication biases may have affected the results of the present scoping review, such as limiting the selection only to papers written in English. Further research may analyze a larger number of studies, also including other decisional tasks, to increase the number of studies considered and to confirm the initial findings of the present review.

Furthermore, it is worth mentioning that the hot and cold EF conceptualization has not commonly been used in literature and the choice to focus the investigation on the relationship between DM and cold EF components within a theoretical framework that considers both hot and cold components could be contradictory. Nevertheless, as stated in the previous paragraphs, we decided to adopt such a theoretical framework to focus on this relationship because, according to recent findings, it is essential to analyze behavioral responses through an integrated approach considering both the cognitive and the affective components (e.g., [26,27]). This is even more necessary when DM under ambiguity and risk are considered, as they are rarely characterized by the absence of emotional or motivational aspects. Furthermore, the definition of hot and cold EFs [30,31,32,33] in DM under ambiguity and risk can be considered as mainly belonging to hot EF components and the relationships with cold EF components are still unclear. Nonetheless, it seems necessary to shed light on these issues, given the possible practical implications that could result. We argue that the adopted theoretical framework, which considers both the influence of the cognitive and the affective dimensions, may offer useful insights to better understand DM mechanisms and the inconsistent findings that sometimes emerge in literature from studies which explored the relationship between DM and EFs.

We are aware that the field is not yet fully defined regarding the considered issues, but we think that this scoping review can contribute to improving the understanding of the topic by mapping evidence published so far concerning the two main tools used to investigate decisional abilities under ambiguity and risks and by suggesting a comprehensive theoretical framework for analyzing EFs, so to suggest the areas to be addressed by future research.

## 5. Conclusions

Through this scoping review we tried to gain a deeper understanding of the relationships between DM—both under ambiguity and risk—and EFs in healthy adulthood. From a broader point of view, the present analysis confirms that the relationship between cold EF components and decisional abilities is the result of a complex interaction, in which cold EFs are essential to a functional decisional process, acting synergically with hot EF components involved in DM. This is possible according to both the cognitive load required by the task and the affective component (elicited by the personal motivation, the ability to predict future outcomes, the recall of previous similar situations, and the processing of contingent information and rewards). Thus, it seems that when little information is available, as in the case of DM under predominant ambiguity, hot EFs play a major role, while cold EFs can contribute to the DM process when more data are available, making choices more functional (as it clearly appears across the blocks of the IGT). Trying to provide a partial answer to Brand and colleagues [10] when they asked: “Is the process for making decisions under ambiguity a pre-requisite for the normal development and execution of decisions under risk, or can the two types of decision-making form independent of each other?” (p. 97), we can maintain that the two processes may be not independent, but they may be mutually modulable according to the degree of “cognitive” information present on a case-by-case basis and the individual’s ability to process information. Thus, the process appears to be highly subjective, depending on the individual’s cognitive functionality together with contextual and task factors. Such claims are consistent with studies that showed neural overlaps of cerebral areas involved during tasks which mostly imply cold EFs and those involving DM. Moreover, it seems consistent also with findings about decisional differences in aging when cerebral changes occur impairing cold EF components and leading to a tendency in OAs to rely more on affective cues (even if cognitive processes can be applied [97]). Consequently, this can lead to risky decisions.

Concluding, although further research is needed to better settle the field, this review confirms the complex nature of DM, highlighting the importance of sustaining cognitive functioning and preventing possible impairments to avoid poor decisions, which can undermine the autonomy and the quality of life, especially during aging. We stressed interesting aspects and possible gaps to answer with further studies. This seems essential to delve into the decisional mechanisms in conditions of ambiguity and risk, which are pervasive in people’s everyday life.

## Figures and Tables

**Table 1:** Characteristics of the samples.

	Country	Size of the Sample	Age Range	Age (Years): Mean (sd)	Education (Years): Mean (sd)
**Iowa Gambling Task**					
Brand et al., 2007 [5]	USA	97	18–65	29.98 (±10.36)	15.99 (±2.35)
Fein et al., 2007 [74]	USA	YAs: 112; OAs: 52	YAs: 18–55; OAs: 56–85	YAs: 37.8 (±10.7); OAs: 73.7 (±7.4)	
Suhr & Hammers, 2010 [75]	USA	57	18–23		
Gansler et al., 2011 [76]	USA	214	18–93	54.7 (±17.4)	14.5 (±2.9)
Xue et al., 2012 [77]	China	430		20.5 (±0.98)	
Reynolds et al., 2019 [78]	USA	56	College students	20.82 (±2.61)	14 (±1.01)
**Game of Dice Task**					
Brand et al., 2008 [79]	Germany	42	19–80	44.45 (±17.46)	
Schiebener et al., 2011 [80]	Germany	80	18–69	33.80 (±14.77)	11.83 (±1.72)
Schiebener et al., 2012 [81]	Germany	100	19–52	23.64 (±6.52)	12.68 (±0.82)
Brand & Schiebener, 2013 [83]	Germany	538	18–80	40.29 (±16.69)	11.81 (±1.66)
Schiebener et al., 2014 [82]	Germany	152	18–75	38.67 (±16.42)	

YAs: younger adults; OAs: older adults. Blank = not specifically reported.

**Table 2:** A summary of the assessment and main results.

	Parameters of the Decisional Task	Cold EFs	Other Cognitive Functions	Assessment	Main Findings
**Iowa Gambling Task**					
Brand et al., 2007 [10]	Net score	(a) flexibility, categorization (b) planning, goal-oriented behavior	(c) verbal intelligence (d) overall intelligence	(a) WCST (perseverative and non-perseverative errors) (b) Tower of Hanoi (c) NART (d) WASI (total IQ, Matrix reasoning, vocabulary)	IGT net score correlated with both perseverative and non-perseverative errors in WCST. WCST perseverative errors predicted the performance of the IGT in blocks 2, 3, 4, and stronger in block 5. No other correlations emerged.
Fein et al., 2007 [74]	Net score	(a) abstraction, flexibility (b) working memory	(c) attention (d) verbal ability (e) psychomotor ability (f) memory (immediate, delay) (g) reaction time (h) spatial processing	(a) Short Categories, Stroop interference score, TMT B, MC: analogies, object match (b) PASAT (c) Stroop Color, MC: numbers forward and reverse, alphabet, word List 1 (d) COWAT, AMNART (e) Trails A, Symbol Digit (f) Rey-Osterrieth Complex Figure, MC: word list, story delay address delay; (g) MC timers (h) Block Design, MC: tic tac, clocks	OAs performed poorer than YAs across the blocks of the IGT. In OAs, IGT performance was associated with abstraction and flexibility, immediate memory, and spatial processing. The IGT performance was predicted by the immediate memory, accounting for 19.9%. In YAs, IGT performance was associated with working memory and psychomotor ability. Working memory and psychomotor ability together explained 12% of IGT performance.
Suhr & Hammers, 2010 [75]	Net score	(a) flexibility, categorization (b) working memory	(c) nonverbal intelligence (d) impulsive sensation seeking and reward driven personality (e) state mood	(a) WCST (perseverative and non-perseverative errors) (b) N-back (omission, commission) (c) Matrix reasoning (WASI) (d) BAS (e) PANAS	Participants who failed in the IGT made more commission errors in the N-back task, performed worse in nonverbal intelligence, and presented significantly less bilateral dlPFC oxygenation.
Gansler et al., 2011 [76]	Net score, choices by deck D minus deck A	(a) flexibility, categorization	(b) fluid intelligence (c) crystallized intelligence (d) visual processing abilities (e) attention	(a) MCST (categories, perseverative errors) (b) Matrix reasoning (WASI), Similarities, Arithmetic (WAIS-R) (c) Information (WAIS-R), NART-R IQ Estimate (d) Block Design, Picture Completion, Digit Symbol (WAS-R) (e) Brief Test of Attention, Digit Span (WAIS-R), CCPT-2	The IGT was associated with general intelligence levels and it was influenced by fluid intelligence. The IGT net score was also directly related to cold EF measures and attention (this one, to a greater extent) in trials 41–100.
Xue et al., 2012 [77]	Net score	(a) working memory (b) inhibition	(c) general intelligence (d) gambler’s fallacy	(a) N-back (accuracy) (b) Stroop Test (c) Raven Advanced Progressive Matrices, WAIS-R (d) Card Guessing Task	There was no association between EF measures and the IGT. The last 60 trials of the IGT were linked to general intelligence. Higher scores in the IGT were also linked to lower gambler’s fallacy bias.
Reynolds et al., 2019 [78]	Net score	(a) flexibility, perseveration	(b) impulsivity (c) risky behavior (d) social desirability (e) memory	(a) WCST (perseverative errors) (b) UPPS-P (c) RBQ (d) MCSDS (e) RDS	No correlation was found between the WCST and IGT. The WCST and IGT were associated with risky behavior assessed through the RBQ: the authors concluded that low cold EFs increase the probability that YAs will engage in risky behaviors.
**Game of Dice Task**					
Brand et al., 2008 [79]	Net score, frequency of choices of alternatives in different risk categories	(a) flexibility, categorization (b) interference susceptibility (c) speed processing (d) verbal fluency	(e) logical thinking abilities (f) verbal intelligence (g) decision making under ambiguity	(a) MCST (categories, total score, number of non-perseverative errors) (b) Interference Trial (WCIT) (c) Word Trial, Color Trial (WCIT) (d)FAS test (e) Reasoning subtest (*Leistungsprüfsystem*) (f) NART (g) IGT	The GDT correlated with cold EFs. In detail, the GDT net score was positively linked to speed processing and negatively to interference susceptibility. The GDT frequency of most disadvantageous choices negatively correlated with the MCST net score and positively with non-perseverative errors and with logical thinking abilities.
Schiebener et al., 2011 [80]	Net score, frequency of choices of alternatives in different risk categories	(a) flexibility, categorization (b) speed of information processing, flexibility	(c) logical thinking abilities (d) decision under probability-based conditions	(a) MCST (categories, total score, number of perseverative and non-perseverative errors) (b) TMT A, TMT B (c) Reasoning subtest (*Leistungsprüfsystem*) (d) PAG	The GDT most disadvantageous choices negatively correlated with the MCST categories, speed of information processing and flexibility, and logical thinking abilities. Whilst it negatively correlated with non-perseverative errors in MCST. The GDT net score was negatively linked with non-perseverative errors MCST. The performance in the GDT was predicted by cold EFs and logical thinking. Participants who made advantageous probability-based decisions in PAG made more advantageous decisions in GDT.
Schiebener et al., 2012 [81]	Frequency of choices of alternatives in different risk categories, number of choices for the more disadvantageous category	(a) flexibility, categorization		(a) MCST (number of non-perseverative errors)	When goal and anchor were provided, the number of GDT most disadvantageous choices correlated with MCST. Participants who performed well in the GDT presented high cold EFs performances.
Brand & Schiebener, 2013 [83]	Net score, frequency of choices of alternatives in different risk categories	(a) flexibility, categorization	(b) logical thinking abilities (c) decisions under ambiguity (administered to 221 out of 538)	(a) MCST (categories, number of perseverative and non-perseverative errors) (b) Reasoning subtest (*Leistungsprüfsystem*) (c) IGT	Age negatively correlated with GDT net scores and positively with GDT frequency of riskiest choices. GDT parameters correlated with MCST and logical thinking abilities. Cold EFs and logical thinking abilities supported advantageous choices in the GDT.
Schiebener et al., 2014 [82]	Net score	(a) interference control (b) flexibility, inhibitory control (c) categorization, rule detection, set maintenance (d) monitoring		(a) CWIT (b) TMT B (c) MCST (number of perseverative and non-perseverative errors) (d) BST	Interference control, flexibility and inhibitory control, MCST, and monitoring correlated with the GDT net score. Interference control and flexibility and inhibitory control predicted the GDT performance, even if a large portion of variance remained unexplained. The GDT net score was negatively linked to age.

BAS: Behavioral Activation Scale; BST: Balanced Switching Task; CCPT-2: Connors’ Continuous Performance Test-2; CWIT: Color Word Interference Test; Cold EFs: cold executive functions; GDT: Game of Dice Task; IGT: Iowa Gambling Task; MCSDS: Marlowe Crowne Social Desirability Scale; MCST: Modified Card Sorting Test; NART: National Adult Reading Task; OAs: older adults; PAG: Probability-Associated Gambling Task; PANAS: Positive and Negative Affect Schedule; RBQ: Risky Behavior Questionnaire; RDS: Reliable Digit Span; TMT: Trail Making Test; UPPS-P: Urgency-Premeditation-Perseverance-Sensation Seeking-Positive Urgency; WASI: Wechsler Abbreviated Scale of Intelligence; WCST: Wisconsin Card Sorting Test; YAs: younger adults.

**Table 3:** Overview of the relationship between the parameters of decisional tasks and cold EFs.

**Iowa Gambling Task—Net Score**						
cold EFs	Brand et al., 2007 [10]	Fein et al., 2007 [74]	Suhr & Hammers, 2010 [75]	Gansler et al., 2011 [76]	Xue et al., 2012 [77]	Reynolds et al., 2019 [78]
flexibility, categorization	perseverative errors: r = −0.351, *p* < 0.001 non-perseverative errors r = −0.322, *p* = 0.001		n.s. ^1^	*p* < 0.001		n.s.
planning	n.s.					
abstraction, flexibility		OAs: r = 0.40, *p* = 0.003				
working memory		YAs: r = 0.32, *p* = 0.001	F = 8.84, ^1^ *p* = 0.004		n.s.	
inhibition					n.s.	
**Game of Dice Task—Net Score**						
	Brand et al., 2008 [79]	Schiebener et al., 2011 [80]	Schiebener et al., 2012 [81]	Brand & Schiebener, 2013 [83]	Schiebener et al., 2014 [82]	
flexibility, categorization	n.s.	non-perseverative errors r = −0.24, *p* < 0.05		categories: r = 0.21, *p* ≤ 0.001 perseverative errors: r = −0.24, *p* ≤ 0.001 non-perseverative errors r = −0.26, *p* ≤ 0.001	perseverative errors: r = −0.19, *p* = 0.05 non-perseverative errors r = −0.25, *p* = 0.01	
verbal fluency	n.s.					
flexibility					r = −0.33, *p* = 0.01	
inhibition/interference control	r = −0.376, *p* < 0.05	n.s.			r = −0.18, *p* = 0.05	
monitoring					1: r = −0.24, *p* = 0.01 2: r = −0.19 *p* = 0.05	
**Game of Dice Task—Number of Most Disadvantageous Choices**						
	Brand et al., 2008 [79]	Schiebener et al., 2011 [80]	Schiebener et al., 2012 [81]	Brand & Schiebener, 2013 [83]	Schiebener et al., 2014 [82]	
flexibility, categorization	total score: r = −0.315, *p* < 0.5 non-perseverative errors: r = 0.319, *p* < 0.05	categories: r = −0.35, *p* < 0.01 non-perseverative errors: r = 0.38, *p* < 0.01	non-perseverative errors: r = 0.57, *p* ≤ 0.001	categories: r = −0.35, *p* ≤ 0.001 perseverative errors: r = 0.35, *p* ≤ 0.001 non-perseverative errors r = 0.38, *p* ≤ 0.001		
flexibility		r = 0.23, *p* < 0.05				

n.s.: no significant relation. ^1^ difference between “failures” and “passers”. Blank = not specifically reported.

## Data Availability

Not applicable.

## Appendix A

### Description of the Decisional Tasks

The Iowa Gambling Task (IGT [10,64]) was formerly designed to assess impairments in everyday decision-making abilities in patients with a lesion located in the vmPFC. So far, it is extensively used to assess DM under ambiguous conditions also in other populations, including individuals without neurological diseases, both in clinical and experimental contexts.

The task requires increasing, as much as possible, an initial amount of money, selecting a card per trial usually from four decks (A, B, C, and D), within 100 total trials. Each deck leads to either monetary wins or losses with different probabilities and frequencies. Specifically, decks A and B are disadvantageous because they present high rewards but also higher penalties, leading the player to an overall loss, while decks C and D are advantageous because they imply small rewards and penalties, leading to an overall gain. Generally, the parameters considered to explore the performance of the task are the total amount of money at the end of the game (either positive or negative), the number of selections from different decks, and the net score (advantageous minus disadvantageous selections) (e.g., [10]). Some researchers divided the task into two main sections, which can represent the two main processes involved. In the first section (i.e., trials 1–40) the probability of loss and win related to each deck is not known to the decision-maker, so he/she chooses the early cards nearly to the chance level (*DM under ambiguity*). During further trials, explicit knowledge about the occurrence of win-and-loss events related to each deck normally begins to arise. Consequently, the decision-maker usually develops a strategy based on previous feedback, showing a tendency to choose advantageous cards and to avoid disadvantageous ones (*DM under risk*). The exact point at which the task switches from ambiguity to risk has not been clearly defined yet, at least in part because it is a gradual and highly subjective process [10,98].

The Game of Dice Task (GDT [66]) is a task widely used to investigate DM under risk. Again, in this task, the goal is to increase the initial amount of money. The decision-maker must bet which face of the die will come up after each roll. He/she can choose either a single number (or face of the die) or a combination of two, three, or four numbers of a die after every throw. There are in total 18 trials, which correspond to the number of throws of the die. For the choice related to each throw, both the rules and the specific wins and losses related to possible choices have been explicitly expressed. To choose adequately, the decision-maker must consider both the possible gain amount and the probability that the choice can occur. Specifically, if the decision-maker chooses a single number, he/she receives the highest monetary win if the chosen number occurs (1:6 chance). Otherwise, if the decision-maker chooses a combination of two numbers, the winning value is a little lower, but the chance to win is a little greater (2:6 chance). Then, the process continues so on for a combination of three numbers, until the decision-maker chooses a combination of four numbers, in which the winning value is the lowest, but the probability of a win is the highest (4:6 chance). Therefore, the choices of one or two numbers are considered disadvantageous or risky, whilst the choices of three or four numbers are defined as advantageous or safe. This task is characterized by explicitness and steadiness over time of the rules. To successfully complete the task, the decision-maker has to consider all information to develop a goal-oriented and successful strategy self-monitoring the decisional process efficiently [66]. Generally, the recorded parameters are the net score (advantageous minus disadvantageous choices) and the frequency of choices of the alternatives in different risk categories (one number = most disadvantageous one; four numbers = most advantageous one) (e.g., [79]).
